# Early Cellular Changes in the Ascending Aorta and Myocardium in a Swine Model of Metabolic Syndrome

**DOI:** 10.1371/journal.pone.0146481

**Published:** 2016-01-14

**Authors:** Rabya Saraf, Thomas Huang, Feroze Mahmood, Khurram Owais, Amit Bardia, Kamal R. Khabbaz, David Liu, Venkatachalam Senthilnathan, Antonio D. Lassaletta, Frank Sellke, Robina Matyal

**Affiliations:** 1 Department of Surgery, Division of Cardiac Surgery, Beth Israel Deaconess Medical Center, Harvard Medical School, Boston, Massachusetts, United States of America; 2 Department of Anesthesia, Critical Care and Pain Medicine, Beth Israel Deaconess Medical Center, Harvard Medical School, Boston, Massachusetts, United States of America; 3 Department of Surgery, Rhode Island Hospital, Brown Alpert School of Medicine, Providence, Rhode Island, United States of America; Virginia Commonwealth University, UNITED STATES

## Abstract

**Background:**

Metabolic syndrome is associated with pathological remodeling of the heart and adjacent vessels. The early biochemical and cellular changes underlying the vascular damage are not fully understood. In this study, we sought to establish the nature, extent, and initial timeline of cytochemical derangements underlying reduced ventriculo-arterial compliance in a swine model of metabolic syndrome.

**Methods:**

Yorkshire swine (n = 8 per group) were fed a normal diet (ND) or a high-cholesterol (HCD) for 12 weeks. Myocardial function and blood flow was assessed before harvesting the heart. Immuno-blotting and immuno-histochemical staining were used to assess the cellular changes in the myocardium, ascending aorta and left anterior descending artery (LAD).

**Results:**

There was significant increase in body mass index, blood glucose and mean arterial pressures (p = 0.002, p = 0.001 and p = 0.024 respectively) in HCD group. At the cellular level there was significant increase in anti-apoptotic factors p-Akt (p = 0.007 and p = 0.002) and Bcl-xL (p = 0.05 and p = 0.01) in the HCD aorta and myocardium, respectively. Pro-fibrotic markers TGF-β (p = 0.01), pSmad1/5 (p = 0.03) and MMP-9 (p = 0.005) were significantly increased in the HCD aorta. The levels of pro-apoptotic p38MAPK, Apaf-1 and cleaved Caspase3 were significantly increased in aorta of HCD (p = 0.03, p = 0.04 and p = 0.007 respectively). Similar changes in coronary arteries were not observed in either group. Functionally, the high cholesterol diet resulted in significant increase in ventricular end systolic pressure and–dp/dt (p = 0.05 and p = 0.007 respectively) in the HCD group.

**Conclusion:**

Preclinical metabolic syndrome initiates pro-apoptosis and pro-fibrosis pathways in the heart and ascending aorta, while sparing coronary arteries at this early stage of dietary modification.

## Introduction

Metabolic syndrome is defined as the cohort of risk factors that increase the likelihood of developing cardiovascular disease (such as coronary artery disease) and type II diabetes mellitus [1]. These physiologic and biochemical changes include abdominal obesity, raised blood pressure, pro-thrombotic state, pro-inflammatory state, insulin resistance, hypertension, and atherogenic dyslipidemia [1]. The inflammation and oxidative stress occurring in these disease states lead to apoptosis and fibrosis at the cellular level in the myocardium, and has been linked to deterioration of endothelial and smooth muscle tissue [2,3]. Simultaneous vascular involvement results in local inflammation, endothelial damage, and atherosclerosis, which cause large artery stiffness, decreased compliance, and coronary artery flow problems [4–6]. While clinically, they are within the spectrum of presentation of metabolic syndrome, the similarity at the biochemical and cellular level has not been conclusively established yet. Determining the exact etiology, timeline of development, and extent of vascular involvement can assist in the identification and grading of myocardial dysfunction.

Furthermore, obesity and related metabolic dysfunction can lead to accelerated hypertension and coronary artery disease, which increase the risk of developing ischemic heart disease and heart failure [2,7]. The aortic changes associated with metabolic syndrome are functionally manifested as reduced vascular compliance and increased after-load burden on the left ventricle (LV). Reduction in LV compliance aggravates myocardial diastolic function while decreased compliance in the ascending aorta abolishes its buffering effect. This not only exposes micro- and macro-vascular beds to pressure variability but also results in end-organ damage [8,9]. The reduction in aortic compliance during metabolic syndrome is a phenomenon that has recently been appreciated [4–6,10]. At the cellular level, apoptosis, fibrosis, and progressive neuropathy in the myocardium are most often implicated as part of the proposed mechanism [11]. There is evidence that the coronary vessels are spared, however, the knowledge of the exact timeline, nature and extent of cellular and biochemical changes in the aorta and myocardium during the early stages of metabolic syndrome remains undetermined. Moreover, whether involvement of the ascending aorta is part of the same patho-physiological process affecting the myocardium is not well established either.

Altered cellular mechanisms in diabetic cardiomyopathy have been identified and provide evidence for the potential development of targeted neuro-hormonal therapy [12]. Similar delineation of a precise mechanism and temporal sequence of vascular and myocardial events during early stages of metabolic syndrome could help in grading and identifying possible therapeutic targets. Such data could also be useful to investigate the aorta as an imaging target early in the development of metabolic syndrome. A large animal model is ideal for such a study since by the time patients present for cardiac surgery, they have already developed advanced aortic atherosclerosis.

In this study, we induced metabolic syndrome through dietary modification for a 12-week period and impaired glucose metabolism in a swine model. The resulting cellular and biochemical changes in the myocardium, aorta, and medium sized arteries during the initial stages of the disease were subsequently analyzed to determine if pathological differences could be observed even if dietary modification was carried out for a short amount of time. Protein expression in the aorta, myocardium, and coronary arteries was quantified for both groups and compared to determine if a high cholesterol diet increased or decreased expression of genes and proteins compared to a normal diet.

## Materials and Methods

### Swine Treatment and Collection

Based on our previous studies involving a similar animal model, we estimated that 8 animals would be required in each group to provide a power of 90% and detect 20% difference in cellular biomarkers and cardiac function with an error of 0.05 [13–15]. Experiments were performed on male Yorkshire swine that were 8 weeks old (Parsons Research, Amherst, MA) as described in our previous publications [13,15,16]. The Institutional Review Board and Institutional Animal Care and Use Committee (protocol #088–2010) at Beth Israel Deaconess Medical Center approved all experiments. Animals were cared for in accordance with the “Principles of Laboratory Animal Care” created by the National Society for Medical Research and the “Guide for Care and Use of Laboratory Animals” (NIH Publication no. 5377–3, rev.1996). A completed ARRIVE guidelines checklist from the National Centre for the Replacement, Refinement & Reduction of Animals in Research is included in S1 ARRIVE Guidelines Checklist [17].

After five days of acclimatization to the environment, the animals were randomized into 2 groups: normal diet (n = 8) and high cholesterol diet (n = 8). The normal diet (ND) group consumed 500 g of commercially available pig chow while pigs in the high-cholesterol diet (HCD) group were fed with 500g of a commercially prepared hypercholesterolemic diet consisting of 75% regular chow, 17.2% coconut oil, 4% cholesterol, 2.3% corn oil, and 1.5% sodium citrate for 12 weeks. This diet is known to induce obesity, hypercholesterolemia, insulin resistance and hypertension [18]. The diet was continued for the duration of the experiment. All animals were kept in cages individually (5 ft. long, 3 ft. wide and 5 ft. high), allowed free access to water and housed in a warm environment throughout the whole experiment.

After 12 weeks of dietary modification, all animals underwent surgery in a large animal operating room in random order. The animals were fasted for 12 hours prior to the procedure. They were given general anesthesia after sedation with Telazol (4mg/kg, intramuscular), followed by endotracheal intubation and ventilation with a volume-cycled ventilator (Dräger, Telford, PA). General anesthesia was maintained with a gas mixture of oxygen at 2 L/min and 2% isoflurane in accordance with laboratory protocols for major animal surgery. The vital signs were recorded intraoperatively and throughout postoperative recovery. Femoral access via a percutaneously placed 4Fr sheath was achieved for arterial access, blood draws and blood pressure monitoring. The heart was exposed via a median sternotomy. Myocardial function was measured using single-sensor pressure catheters (Millar Instruments, Houston, TX) and sonomicrometer crystals (Sonometrics Corp. London, ON, Canada). The mean arterial pressure (MAP), left ventricular pressure developed, positive dP/dt and negative dP/dt were measured for ten sequential beats. Cross sections at the mid-papillary levels were taken for analysis for myocardial molecular experiments.

Coronary blood flow: X-ray coronary angiography was carried out to compare the coronary blood flow by using TIMI score (Thrombolysis in Myocardial Infarction) from 0–3 for right coronary artery (RCA) and left anterior descending artery (LAD). After functional analysis and coronary angiography, the hearts, aorta and coronary arteries were harvested and frozen in liquid nitrogen for further analysis or fixed in 10% formaldehyde and paraffinized for immunohistochemistry.

Height and weight were measured after induction, before the beginning of the procedure. Blood samples were collected after femoral sheath placement and spun for serum and plasma analysis.

### Terminal deoxynucleotidyl transferase dUTP nick end labeling (TUNEL) Assay

Sections were deparaffinized in xylene and rehydrated through a series of decreasing alcohol solutions. Sections were subsequently treated with Proteinase K (Sigma-Aldrich, St. Louis, MO) for fifteen minutes at room temperature followed by incubation with 3% hydrogen peroxide for ten minutes. A Streptavidin/Biotin Blocking kit (Vector Laboratories, Burlingame, CA) was used to quench the endogenous biotin activity. Afterwards, a biotin-dUTP labeling reaction mixture (Roche, Penzberg, Germany) was added to the sections and incubated for 1 hour at 37°C. Sections were washed with stop wash buffer (300 mM NaCl and 30 mM Sodium Citrate) for 10 min at room temperature. After rinsing sections in TBS, sections were incubated with HRP-streptavidin conjugates in TBS for 30 min at room temperature. Apoptotic nuclei were visualized using a DAB substrate kit (Vector Laboratories, Burlingame, CA). Finally, slides were counterstained with hematoxylin, dehydrated through graded alcohols, cleared in xylene and mounted with Permount (Fisher Scientific, Asheville, NC).

### Immunofluorescence labeling of PGP9.5 and beta-myosin

The paraffin sections were deparaffinized, rehydrated and treated with 10 mM Sodium Citrate pH 6.0 for antigen retrieval. The sections were then incubated with 1 mg/ml sodium borohydride (ICN chemicals) for 5 minutes at room temperature. After three washes with TBS, the sections were incubated with 5% normal donkey serum (Jackson ImmunoResearch Lab Inc, West Grove, PA) for an hour at room temperature. Slides were then incubated overnight with mouse anti-PGP9.5 (Ubiquitin-protein hydrolase involved both in the processing of ubiquitin precursors and of ubiquitinated proteins, specifically present in neurons and cells of the diffuse neuroendocrine system.1:100, Cedarlane) at 4°C. The slides were washed three times and incubated with Dylight 549 Donkey anti- mouse secondary antibodies (Jackson ImmunoResearch Lab, 1:200) for 90 minutes at room temperature. Sections were then counterstained with Hoescht 33342 nuclear dye (Life Technologies, Woburn, MA) before they were mounted with Prolong Gold anti-fade mounting media (Life Technologies, Woburn, MA). Confocal images were taken using a Zeiss LSM510 Meta confocal system using Zeiss LSM510 image acquisition software. The images were taken using a 20 /0.8 Plan-Apochromat objective and a 40 /1.3 Oil Plan-Apochromat objective.

### Sirius Red Collagen IHC staining

Samples were deparaffinized and hydrated. Nuclei were stained with Weigert's hematoxylin for 10 minutes and washed in water. Slides were stained with picro-sirius red (0.5 g Sirius red powder (F3B), 500 ml picric acid (saturated) solution) for 1 h and washed twice with acidified water (5 ml glacial acetic acid, 1 l distilled water). Water was removed through blotting with vigorous shaking. Slides were dehydrated three times in 100% ethanol, cleared in xylene, and mounted with Permount (Fisher Scientific, Asheville, NC).

Protein Carbonyl Content Assay (Nitrosyl Test)

Experiment was conducted per the instructions on the OxyBlot Protein Oxidation Kit (Millipore, Billerica, MA).

### Immunoblotting

Whole cell lysates were made from the harvested arteries (aorta, LAD, and RCA) and normal myocardium. Protein concentrations were determined through a Bicinchoninic acid assay (BCA Assay) (Fisher Scientific, Asheville, NC). Fifty micrograms of protein were loaded in each lane and GAPDH (Cell Signaling Technologies, Danvers, MA) was used to normalize the results. Samples from each group were separated using a 4–12% gradient SDS-PAGE (Life Technologies, Grand Island, NY) and transferred to PVDF membranes (Millipore, Billerica, MA) using a semi-dry transfer cell (Bio-Rad Transfer Blot, Hercules, CA).

Blots were probed for pro- and anti-apoptotic markers cleaved caspase-3, caspase-3, phospho-Akt, Bcl-xL, p38MAPK and Apaf-1 (Cell Signaling Technologies, Danvers, MA). We also examined fibrotic factors MMP-9, Smad3, and TGF-β (Cell Signaling, Danvers, MA), oxidative stress related factors MnSOD (R&D Systems, Minneapolis, MN), ADIPOR1 (Biorbyt, Cambridge, UK), eNOS, (Cell Signaling Danvers, MA) and NF-κβ (Abcam, Cambridge, MA). Primary antibodies were incubated in 4°C in 3% BSA overnight and washed in PBST before incubating in their corresponding anti-mouse or anti-goat secondary antibodies (Cell Signaling Technologies, Danvers, MA) in 3% BSA for one hour at room temperature. Immune complexes were visualized with an enhanced chemiluminescence detection system (Thermo Scientific, Amersham, Piscataway, NJ). All the blots were normalized against the background and with GAPDH protein.

### Animal Data

Height and weight were measured at the end of each surgery. The glucose tolerance test was conducted at the 0, 30 and 60-minute intervals after 0.5g/kg of dextrose infusion. The serum was obtained and sourced to a laboratory for assessment of lipid profile by analysis of the ratio between plasma proatherogenic apoB and antiatherogenic apoA-1. ApoB/ApoA ratio (LDL/HDL ratio) is a greater risk indicator with greater predictive value than isolated parameters) and was used as described by Walldius et al [19].

### Statistical Analysis

All results were expressed as standard deviation. Probability values of less than or equal to 0.05 were considered significant. Myocardial function was quantified via measurements of mean arterial pressure, left ventricle systolic function, left ventricle diastolic function (-dp/dt) and fractional shortening (a measure of left ventricular thickening). The western blots were quantified with ImageJ (National Institutes of Health, Bethesda, MD) and analyzed by one-way ANOVA and multiple t-test comparisons (Statistical Package for Social Sciences Version 21, Chicago, IL) after normalization.

## Results

### Baseline Characteristics

**Fig 1:**
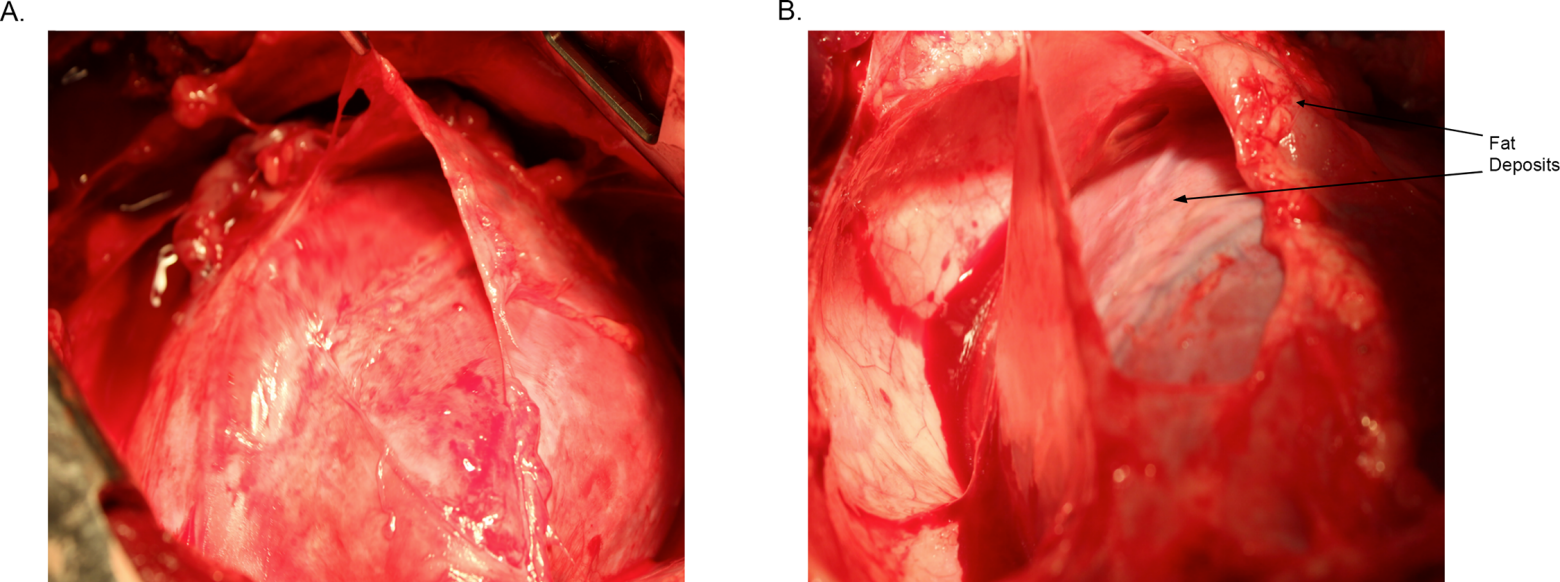
Fat deposits on heart tissue and illustration showing site of tissue excision. Representative picture of heart and surrounding pericardium and epicardium from A) normal diet (ND) and B) high cholesterol diet (HCD) groups. Notice the fat deposits in the HCD heart both on the pericardium and epicardium.

All animals (n = 8/8) were included in the analysis. There was no significant difference in the body mass index (BMI) between the groups at the beginning of the experiment (25.8 ± 6 kg/m^3^ vs. 27.3 ± 3 kg/m^3^, p = 0.4). After 12 weeks of modified diet, the BMI was significantly higher in the HCD swine compared to ND swine (40.4 ± 2.7 kg/m^3^ vs. 32.6 ± 3.4 kg/m^3^, p = 0.002). In addition, despite similar fasting blood glucose levels in both groups, the blood sugar levels 30 minutes after intravenous dextrose challenge was significantly elevated in the HCD group as compared to the normal diet group (157 ± 7.7 mg/dL vs. 113 ± 5.9 mg/dL p = 0.001). The ratio of Apo B100/ApoA (LDL/HDL ratio, greater risk indicator with greater predictive value than isolated parameter) was 0.03 ± 0.05 in ND, and 0.25 ± 0.4 in HCD (p = 0.15) (Millan 2009). Global histology revealed increased fat deposits around the epicardium and pericardium of HCD treated pigs compared to the ND group (Fig 1).

### Apoptosis

#### Aorta, LAD, and RCA

**Fig 2:**
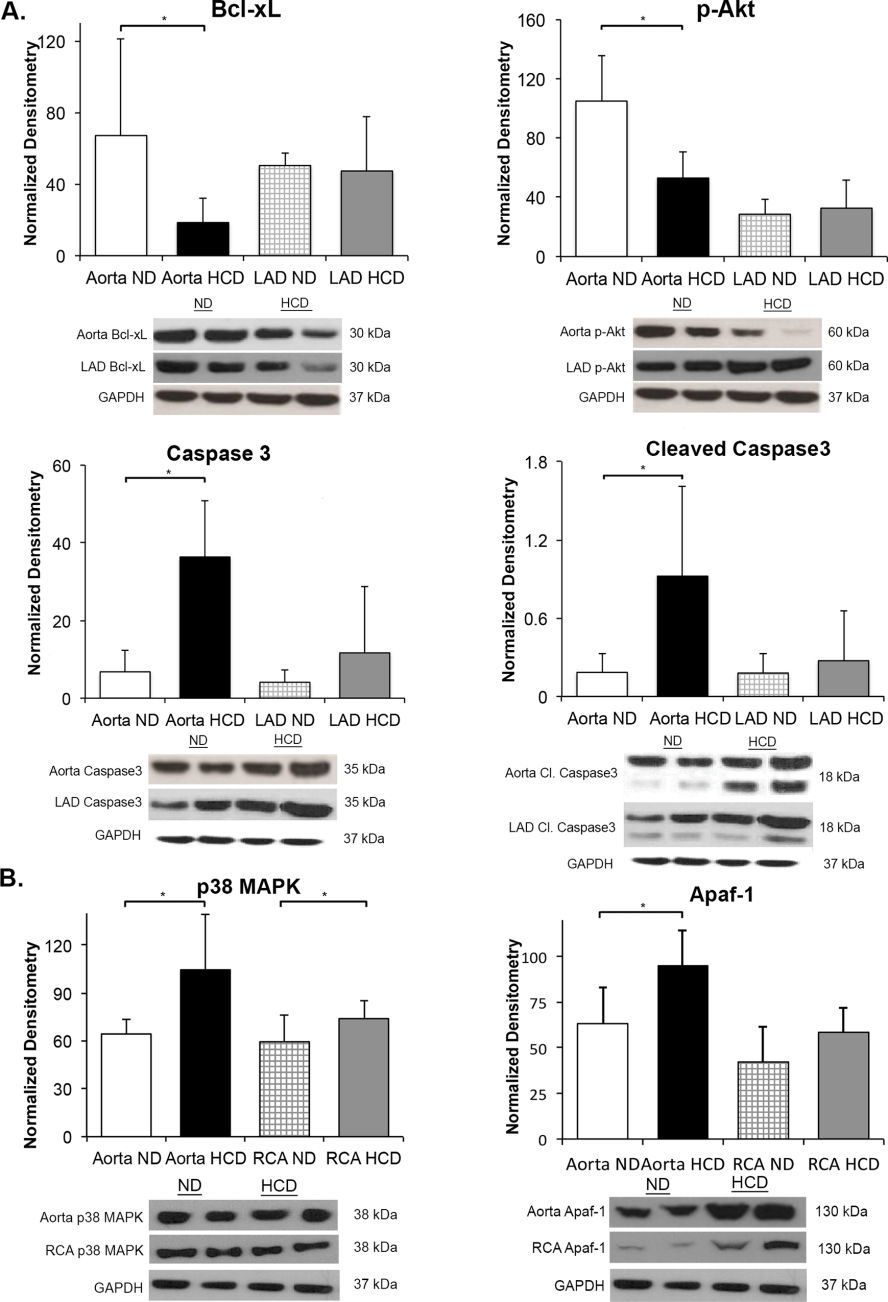
Protein expression of pro- and anti- apoptotic proteins in the aorta, LAD, and RCA for both the ND and HCD groups. (A) There is significant increase in Bcl-xL, Caspase3 and cleaved Caspase 3 in the aorta in HCD group. Significant change is not observed in the LAD in HCD. (B) There is significant increase in p38MAPK in both aorta and RCA in HCD group suggesting altered myocardial remodeling. The levels of Apaf-1 are significant only in the aorta. (*p-value<0.05).

The levels of anti-apoptotic markers Bcl-xL and p-Akt were significantly decreased in the aorta of the HCD group (p = 0.05 and p = 0.007, respectively) whereas there was no significant difference in the LAD HCD and ND groups (p = 0.74 and p = 0.94, respectively). The expression of p38MAPK was significantly increased in the aorta and RCA (p = 0.037 and p = 0.04, respectively) in the HCD group. While the expression of pro-apoptotic factors Caspase3, cleaved Caspase3, and Apaf-1 were significantly increased in the aorta of the HCD group (p = 0.009, p = 0.007 and p = 0.04, respectively) there was no such difference seen in the LAD and RCA between the ND and HCD groups (Fig 2).

#### Myocardium

**Fig 3:**
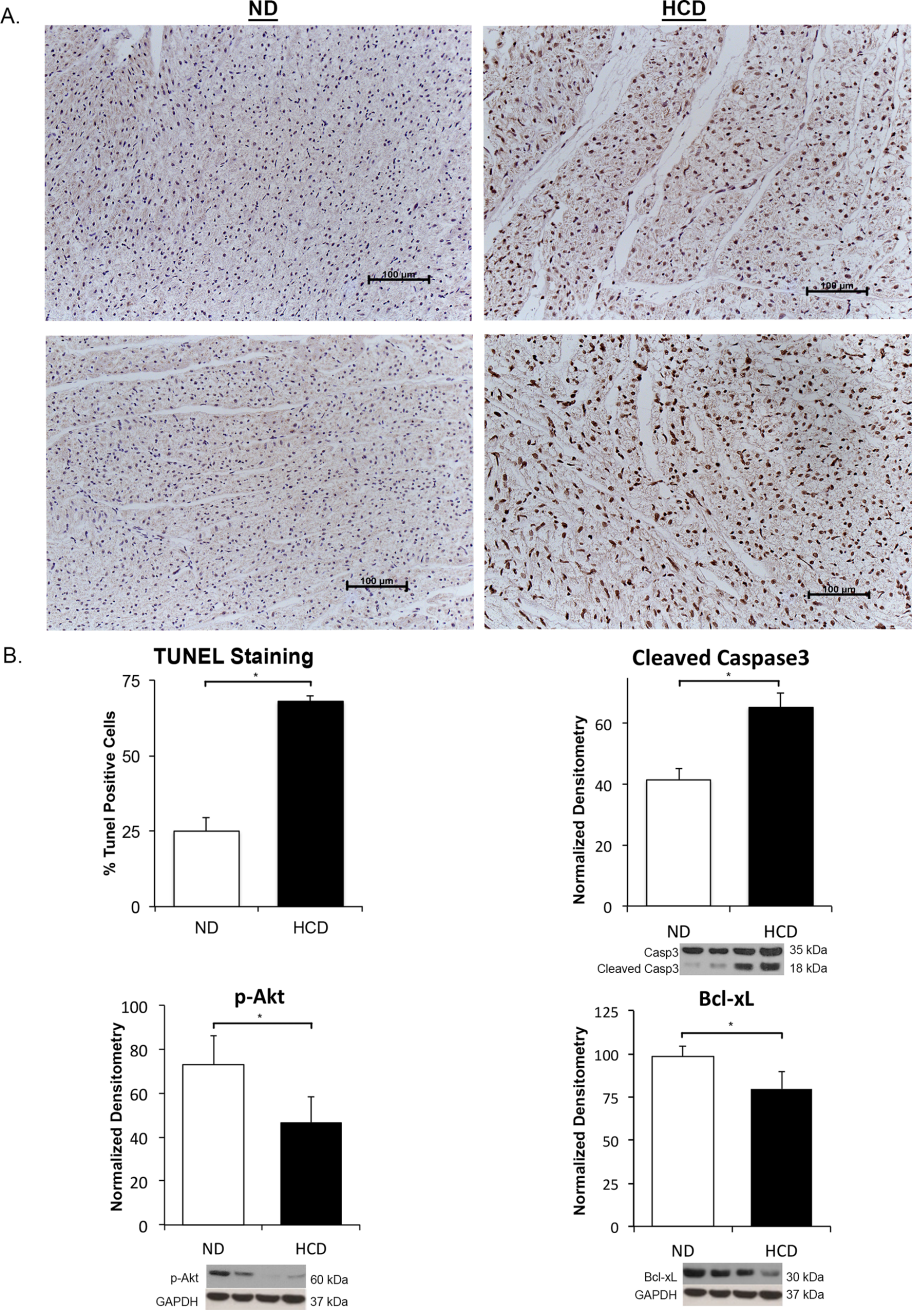
Apoptosis in the myocardium. There is significantly increased TUNEL staining in HCD group representing apoptosis. The markers for apoptosis are significantly altered in the myocardial tissue. p-Akt is significantly decreased, which may be involved in apoptosis and leads to up-regulation of p38MAPK leading to pathological remodeling. (*p-value<0.05).

The myocardium from the HCD group revealed significantly increased pro-apoptotic activity on immunohistochemistry with TUNEL positive cells and on Western blot with increased expression of cleaved Caspase3 (p = 0.004 and p = 0.03, respectively). There was a simultaneous significant decrease in anti-apoptotic proteins p-Akt and Bcl-xL in the myocardium of the HCD group (p = 0.002 and p = 0.01, respectively) (Fig 3).

### Fibrosis

#### Aorta and LAD

**Fig 4:**
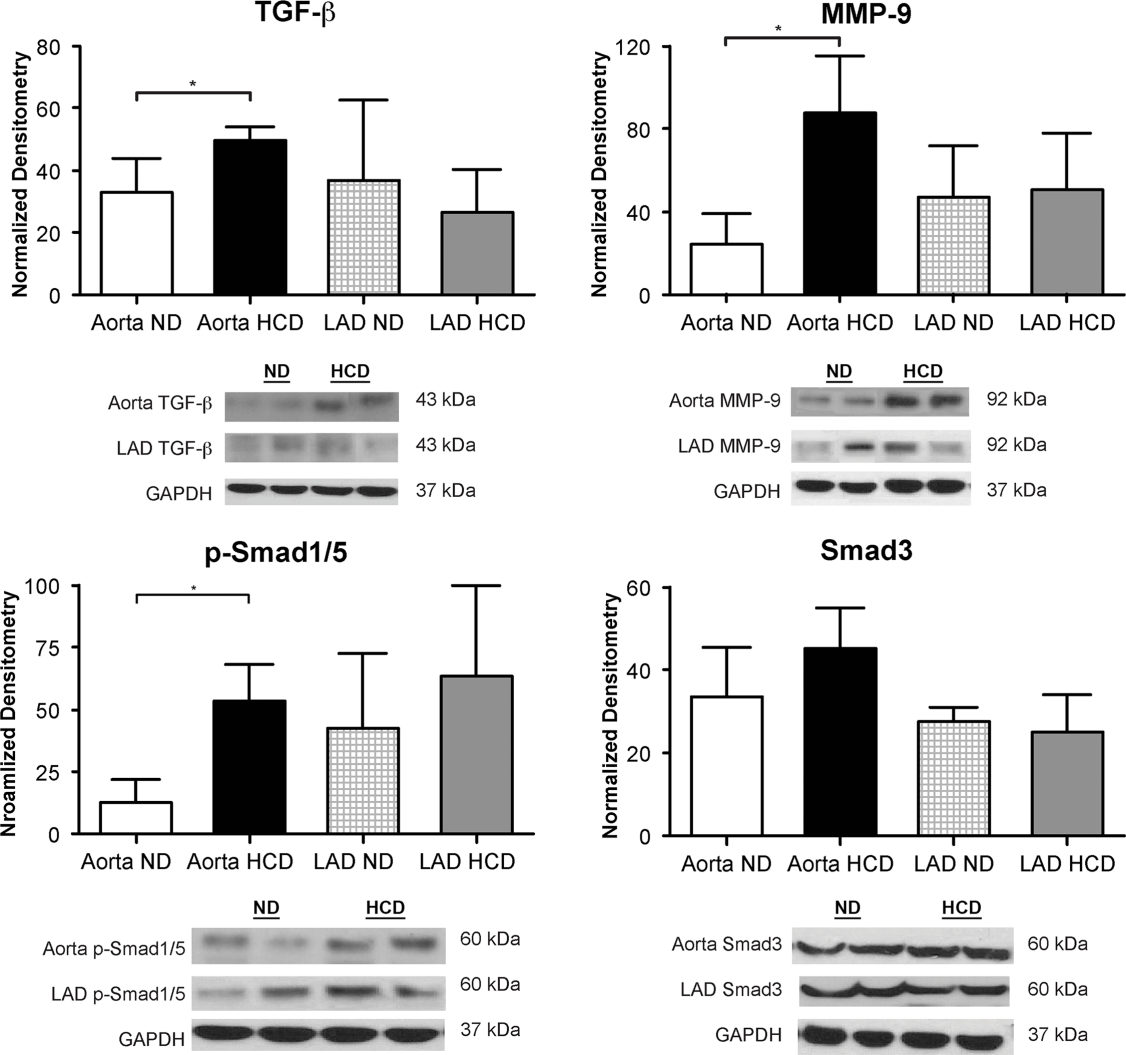
Immunoblotting data for markers of fibrosis in aorta and LAD. The markers for fibrosis and MMP-9 are markedly increased in the ascending aorta of the HCD group. There is no fibrosis in the LAD in HCD group. (*p-value<0.05).

Western blotting revealed increased levels of TGF-β and MMP-9 in the aorta of the HCD group (p = 0.01 and p = 0.005, respectively). p-Smad1/5, an intracellular protein involved in transducing signals, was up-regulated in the HCD group (p = 0.03). There was no significant difference between ND and HCD for Smad 3, also an intracellular protein involved in transducing signals (p = 0.07). Similarly, differences in TGF-β, MMP-9, p-Smad1/5 and Smad3 were not observed in the LAD between the two groups (p = 0.22, p = 0.27, p = 0.24 and p = 0.50, respectively) (Fig 4).

#### Myocardium

**Fig 5:**
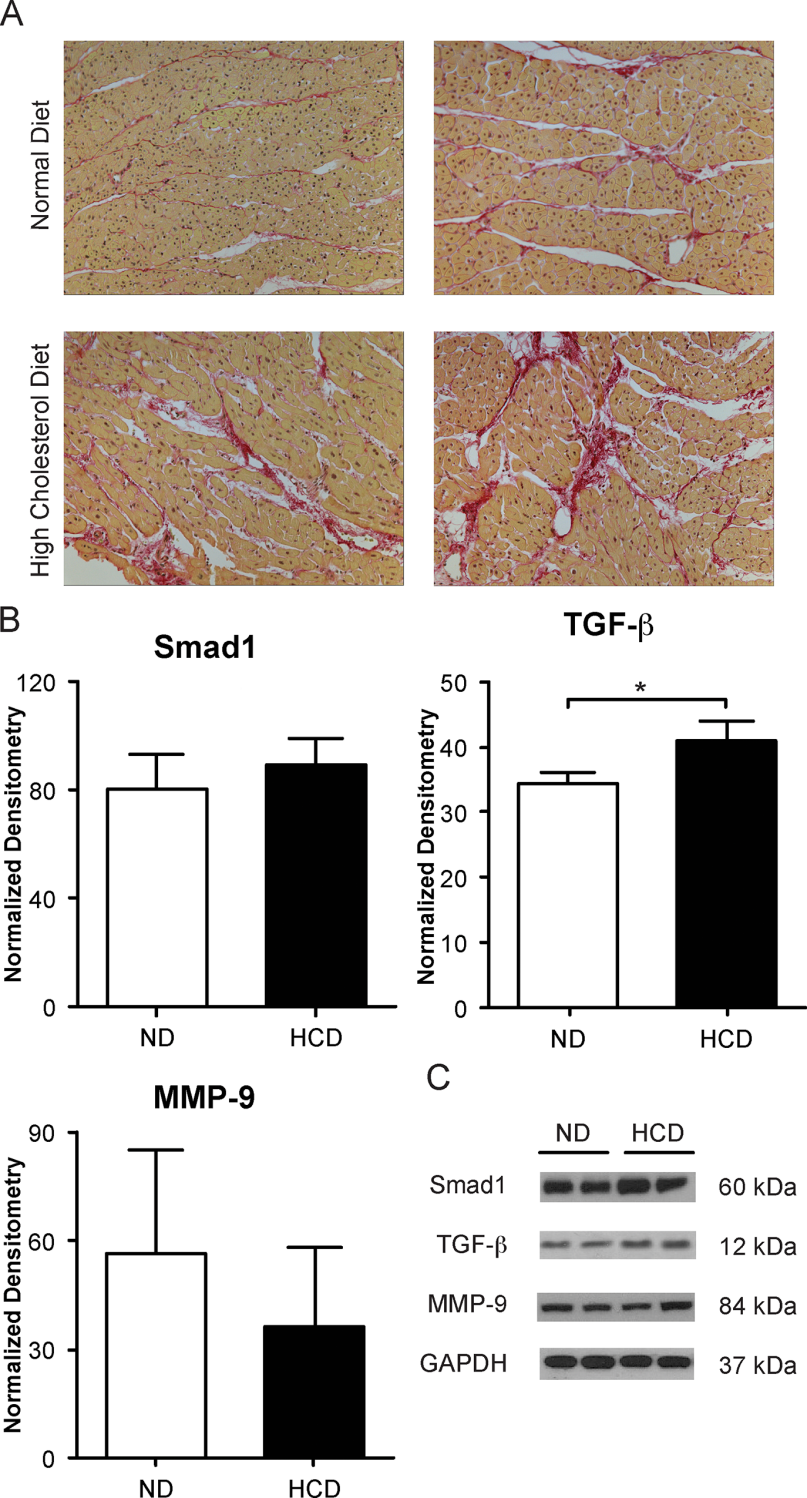
Fibrosis in the myocardium. (A) Immunohistochemistry of fibrosis through Sirius red staining taken at 20X (Bright field images seen here). Staining shows greater amounts of fibrosis in the myocardium of the HCD group. (B) Immunoblotting of pro-fibrotic marker Smad1, transforming growth factor-β (TGF-β) and matrix metalloproteinase-9 (MMP-9). (C) Immunoblots of the corresponding proteins. (*p-value<0.05).

Bright field imaging from sirius red staining showed increased fibrosis in the HCD group compared to the ND group. The levels of TGF-β were significantly increased in the HCD group (p = 0.01) while Smad1 and MMP-9 were not significantly changed in the HCD group (p = 0.18 and p = 0.23, respectively) (Fig 5).

### Neurovascular Markers

**Fig 6:**
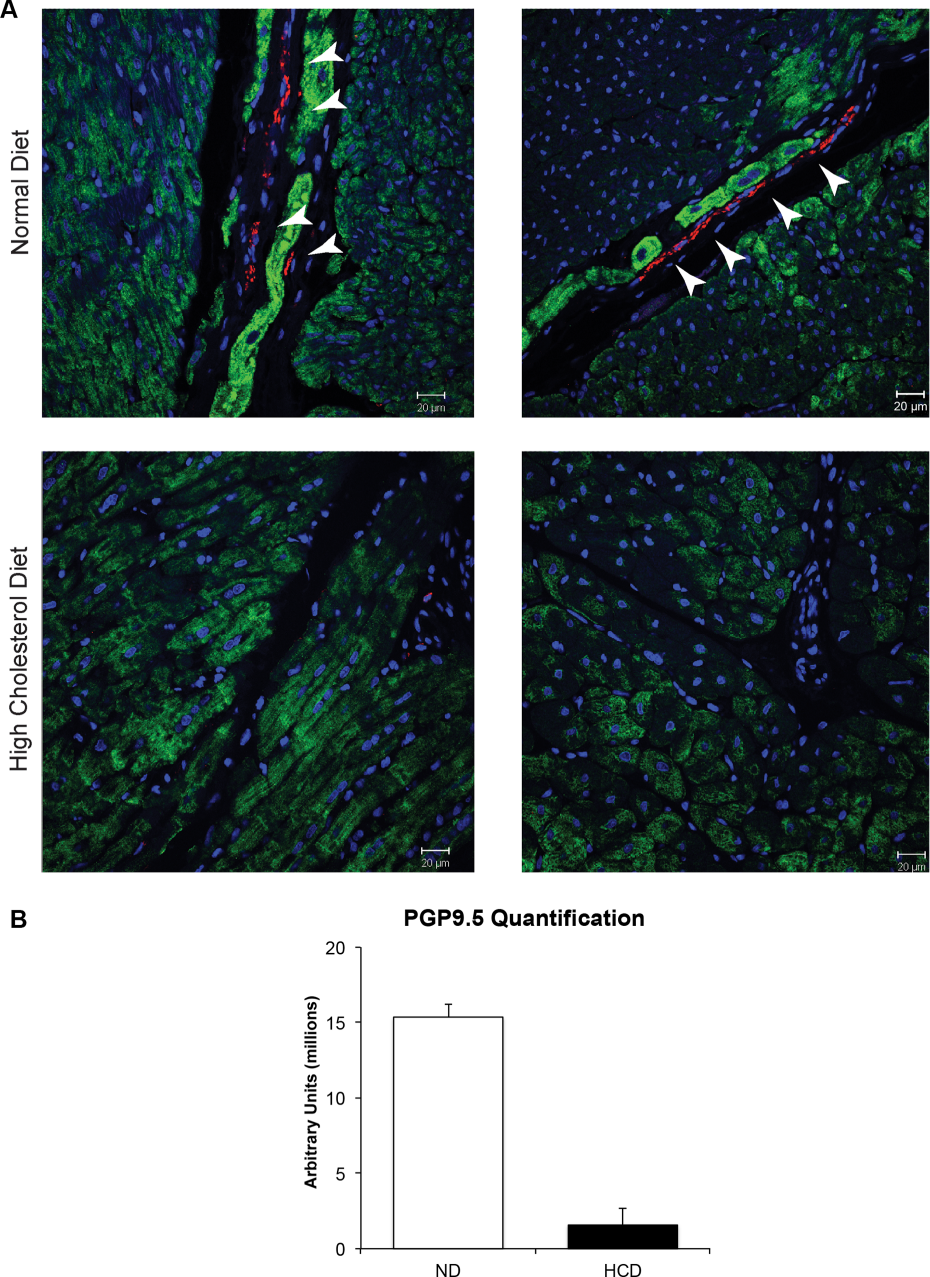
Expression of neurovascular markers in the myocardium. (A) The slides from myocardial sections from normal (ND) and high cholesterol diet (HCD)-treated swine for PGP9.5. (B) PGP9.5 quantification showing a significant decrease in sympathetic nerves in the high cholesterol diet treated pigs. (*p-value<0.05).

PGP9.5 (ubiquitin carboxy-terminal hydrolase L1), a specific marker of neurons that makes up 1–2% of soluble proteins in neurons, was significantly decreased in the HCD group compared to the control (p = 0.003) (Fig 6).

### Oxidative Stress

#### Aorta and LAD

**Fig 7:**
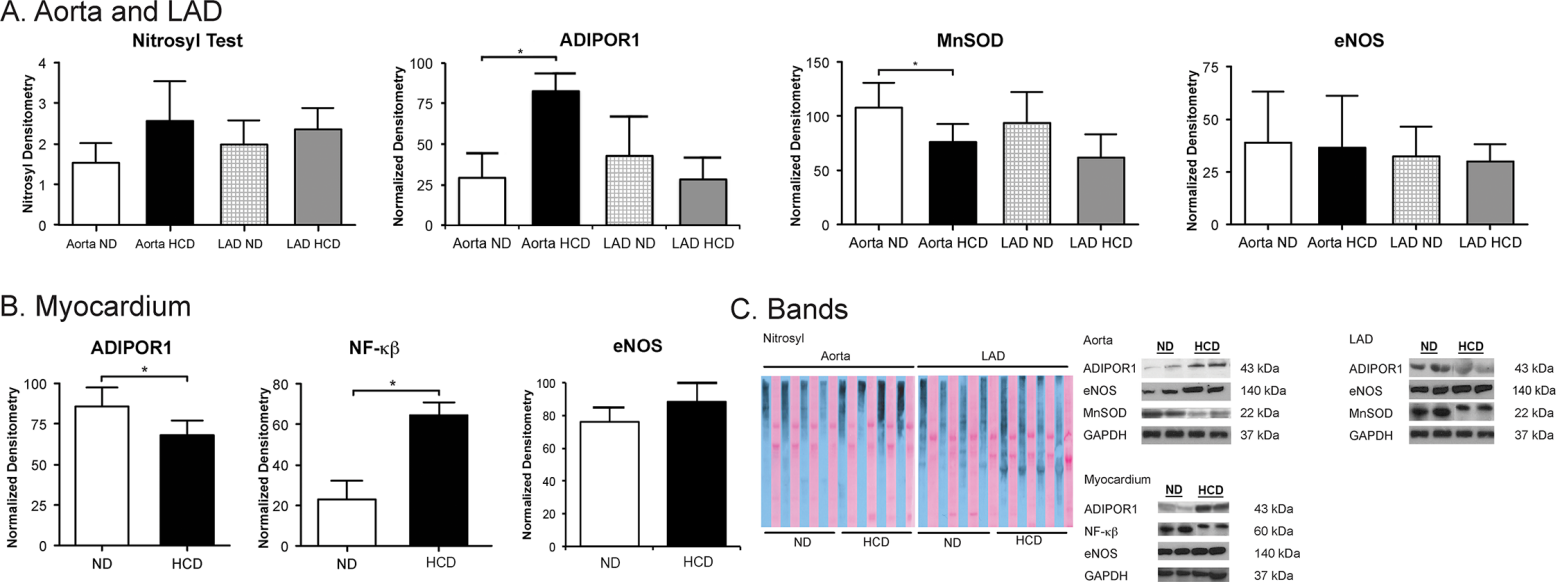
Expression of oxidative stress, anti-oxidative stress and inflammatory markers. (A) Aorta and LAD; (B) Myocardium. There is a trend towards increased nitrosyl oxidation in aorta and LAD HCD groups. There are significantly increased levels of ADIPOR1 in the HCD group aorta, while significantly decreased levels in the myocardium of HCD animals. The anti-oxidative marker MnSOD is significantly down-regulated in HCD aorta, while trending down in LAD. There is significantly increased inflammatory marker NF-κβ in the myocardium. The levels of eNOS are not changed in aorta, LAD, and myocardium. (C) Nitrosyl test and immunoblots of the corresponding proteins. (*p-value<0.05).

There were insignificant elevations in the levels of protein carbonyl content in the aorta of the HCD group on nitrosyl test (p = 0.29). Anti-oxidative stress marker ADIPOR1 was significantly increased in the aorta of the HCD group (p = 0.018), while no significant change in eNOS levels (p = 0.61) was seen. The levels of MnSOD, an important anti-oxidant, were significantly lower in the aorta of the HCD group (p = 0.003). There were no significant changes observed in the LAD of the HCD group as compared to the ND group for nitrosyl test, ADIPOR1, MnSOD or eNOS (p = 0.26, p = 0.55, p = 0.53 and p = 0.38 respectively) (Fig 7A).

#### Myocardium

ADIPOR1 was significantly decreased in the myocardial tissue of the HCD group (p = 0.018). The oxidative stress and inflammation marker NF-κβ was significantly increased in the HCD group myocardium (p = 0.001). The levels of eNOS were not significantly different between the two groups (p = 0.6) (Fig 7B).

### Myocardial Functional Measurements

**Fig 8:**
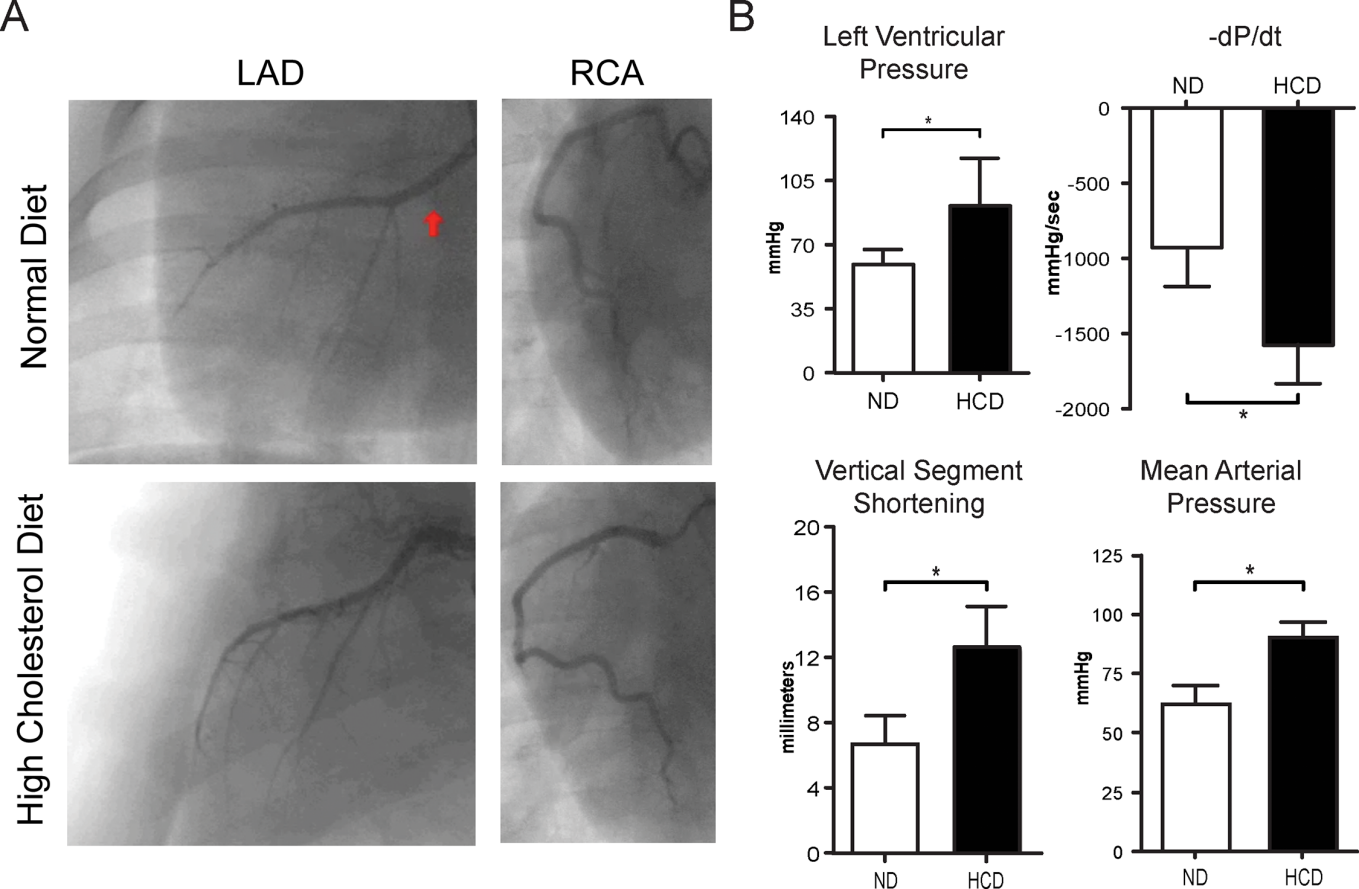
Hemodynamic data for swine. (A) Coronary angiography in high-cholesterol diet and normal diet pigs demonstrates no differences in coronary flow between the two groups and is confirmed by TIMI-flow score (B) Hemodynamic data of swine. Invasively driven left ventricular functional data comparing–dp/dt, left ventricular end systolic pressure, mean left ventricular pressure and vertical segmental shortening. (*p-value<0.05).

There was no difference in blood flow as measured by TIMI score (TIMI score 3) in the LAD and RCA territory in ND and HCD groups and coronary angiogram showed normal flow across both groups. There was increased left ventricular end systolic pressure (p = 0.05). Even though there was no change in +dp/dt (p = 0.2) there was significant increase in–dp/dt (p = 0.007). The mean arterial pressure and vertical segment shortening were significantly increased in the HCD group (p = 0.024 and p = 0.016 respectively) (Fig 8).

## Discussion

The results of our study have demonstrated that simultaneous biochemical and cellular changes are exhibited in the myocardium and the ascending aorta in response to three-month exposure to a high-cholesterol diet in a young swine model. Interestingly, these cellular changes were not observed in the coronary arteries at the end of the experiment. In terms of physiologic changes, the induced metabolic syndrome resulted in elevated peak systolic ventricular pressure and increased–dp/dt with no significant change in +dp/dt. There was evidence of increased inflammatory markers without any significant evidence of oxidative stress at this early stage of subclinical metabolic syndrome. The findings of our study are pertinent in that they show that a relatively short duration of diet-induced subclinical metabolic syndrome is able to elicit ultra-structural changes in the myocardium and the aorta while sparing the coronary arteries. While an exact cause-and-effect relationship is difficult to establish at this point, our findings raise the possibility of identifying specific biochemical pathways that underlie metabolic syndrome-associated large artery vasculopathy.

The pathological remodeling in the myocardium and aorta has been described in previous literature as resulting from an adaptive response to hypertension, hyperglycemia, hyperlipidemia, and inflammatory molecules and hormones [20,21]. These cardiac processes are involved in regulating many intracellular signaling pathways in cardiomyocytes [22]. Mitogen-activated protein kinases (MAPKs) are one particular type of intracellular kinases that were shown to play a role in heart damage during metabolic syndrome (22). Furthermore, remodeling of the cardiovascular system due to dietary modification has been seen in adult swine as early as six months post-modification, resulting in vascular smooth muscle cell proliferation and increased extracellular matrix [23]. Micro-vascular remodeling is multifactorial and involves proliferation of vascular smooth muscle cells, deposition of advanced glycation end-products on the arterial wall, and activation of TGF-β, which has been suggested as a causative mechanism responsible for the crosslinks in collagen leading to stiffness [2,4,5,7,24–29]. The apoptotic marker p38 mitogen-activated protein kinases (p38MAPK) has been known to play a role in metabolic syndrome-associated myocardial remodeling in conjunction with hypertrophy, fibrosis, and apoptosis. Our present study suggests that the activation of p38MAPK in the ascending aorta might be involved in activating pro-fibrotic and pro-apoptotic pathways.

Protein kinase B, also known as Akt, is a multifunctional protein kinase that is implicated in such processes as glucose metabolism, apoptosis, cell proliferation, transcription, and cell migration. Akt signaling has also been associated with physiological cardiac growth and remodeling [30]. The significant decrease seen in our early model of metabolic syndrome is of importance because decreased levels of Akt can actually revert pathological remodeling, resulting in apoptosis and fibrosis [30]. Akt also actively suppresses signaling pathways that are involved in pathological remodeling of the heart, such as the p38MAPK and extracellular signal-regulated kinases (ERK) pathways [31]. The significant activation of p38MAPK in aortic tissue seen in our results may explain the pro-fibrotic and pro-apoptotic signals. The associated increase in NF-κβ leads to further increase of the inflammatory response. The significant decrease in Akt is an important finding since it is able to suggest the change in signaling from cell survival to apoptosis at an early stage of metabolic syndrome in young swine. Up-regulation of the p38MAPK pathway and down-regulation of the Akt pathway are both significant factors involved in causing early changes in the heart tissue of diabetic patients [32].

Our study highlights the cellular pathways that contribute to vasculopathic changes and suggests that these changes can be initiated as early as three months after dietary modification in young swine that have pre-metabolic syndrome. This may also contribute evidence to the possibility of developing targeted anti-inflammatory therapy for metabolic syndrome-related vascular pathology.

The presence of excess epicardial visceral fat causes an increase in inflammatory cytokines such as leptin and adiponectin receptor 1 (ADIPOR1) that are secreted by the surrounding adipose tissue [33]. Adiponectin receptors, which are transmembrane proteins, mediate fatty acid oxidation and glucose uptake by adiponectin. Examination of heart tissue after completion of the experiments revealed fat deposits on the heart (Fig 1). In addition, the altered levels of ADIPOR1 in the myocardial and aortic tissue in our study may be reflective of the altered activation of adipose tissue cytokines that result in impaired fatty acid metabolism [34].

There is increased recognition of aortic involvement early on in metabolic syndrome, leading to aortic stiffness [10]. Aortic stiffness and atherosclerosis have been shown to be independent predictors of cardiovascular morbidity and mortality and of stroke [35]. However, the pathophysiology of aortic stiffness is incompletely understood and appears to be multifactorial [8,36,37]. Initially, the proximal aorta develops stiffness, which can be measured with magnetic resonance imaging (MRI). The physiological consequences of aortic stiffness include increased pulse pressure and reduced distensibility. Since by the time most patients present for cardiac surgery they already have advanced atherosclerosis, the early cellular and biochemical changes are difficult to establish in a human model. While our findings may not be entirely representative of human processes, they offer insight into the cellular pathology underlying the development of aortic stiffness in metabolic syndrome in a similar model.

Interestingly, our findings closely mirror those seen in age-associated vasculopathy. At the cellular level there is evidence of altered vascular structural matrix proteins and activation of matrix metalloproteinases (MMPs), inflammation, endothelial damage, and altered mitochondrial bioenergetics [38]. Activation of pro-apoptotic and pro-fibrotic cellular signaling pathways due to endoplasmic reticulum stress has been associated with aortic stiffening and vascular smooth muscle changes in chronic hypertension [39]. Similarly, endothelial damage with apoptosis has been seen in cells exposed to hyperglycemia [40]. The observed selective sparing of medium sized coronary arteries is similar to what is seen in age-related deterioration where the aorta undergoes changes but the medium sized arteries are spared. Based on evidence available in literature and the findings of our own study, it appears that in terms of deterioration and biochemical and cellular changes, metabolic syndrome is analogous to an accelerated aging process [41]. The observed changes seen in a young swine model with only three months of dietary modification suggest that the responsive signaling pathways are activated early on, leading to apoptosis and fibrosis in the myocardium and aorta while sparing the coronary arteries. A study conducted by Trask and colleagues pointed to changes in the LAD arteries after six months of dietary modification, suggesting a timeline for the occurrence of these events in the coronary arteries and the aorta [23]. Given these findings from Trask et al, our experiments could contribute to developing a tentative timeline for cellular events in the coronary arteries [33].

In our model, there seems to an altered cellular oxidative environment as partially evidenced by changes in ADIPOR1 levels, mitochondrial manganese superoxide dismutase (MnSOD) and nitrosative stress. There was also increased activity of inflammatory markers (TGF-β and NF-κβ), collagenase activity, and extracellular matrix with resultant fibrosis. The fibrosis and apoptosis in the ascending aorta could possibly be the etiological mechanism for the aortic stiffness that develops in chronic metabolic syndrome. Stiffening of the aorta impairs the cushioning function of the arteries and results in increased systolic blood pressure and decreased diastolic pressure [42]. This impaired relaxation may precede eventual cardiovascular disease that causes left ventricular hypertrophy, diastolic dysfunction and congestive heart failure [43,44]. Above all, a raised pulse pressure secondary to fibrosis-induced aortic stiffness results in arterial remodeling and plaque formation. TGF-β is not only involved in apoptosis of myocytes, but also the alteration of vascular endothelium and activation of matrix metalloproteinases (MMPs), that modify interstitial matrix in the aorta [45,46]. Notably, TGF-β has been implicated as an important mediator of kidney disease in diabetes by regulating SMAD and MMP pathways [46].

## Limitations

The major limitation of this study was the short period of time that the swine model was investigated for metabolic syndrome-inducing high cholesterol diet. The brevity of this investigation precluded a more complete delineation of the timeline of events in the development of vascular pathology following occurrence of metabolic syndrome. However, the fact that pathologic changes were observed within the three-month experimental window signifies the relatively rapid nature of the cellular response to oxidative stress in the large arteries due to a high cholesterol diet and subsequent metabolic syndrome.

## Conclusion

Subclinical metabolic syndrome in a swine model leads to fibrosis and apoptosis in the aorta and myocardial tissue while sparing the coronary arteries at an early stage of dietary modification. These changes occur simultaneously in the myocardium and the aorta, and may be similar to those observed in age-related vascular damage.

## Supporting Information

S1 ARRIVE Guidelines Checklist Completed “The ARRIVE Guidelines Checklist” for reporting animal data in this manuscript. (PDF)
